# Recatest—A Technique for Qualitative and Quantitative Assessment of Deferment and Degraded PVD Coatings and CVD Layers in the Deformed Area in the Scratch Test

**DOI:** 10.3390/ma14102625

**Published:** 2021-05-17

**Authors:** Piotr Domanowski, Marek Betiuk

**Affiliations:** 1 Faculty of Mechanical Engineering, UTP University of Science and Technology, Al. Prof. S. Kaliskiego 7, 85-796 Bydgoszcz, Poland; 2 Lukasiewicz Research Network, Institute of Precision Mechanics, ul. Duchnicka 3, 01-796 Warszawa, Poland; marek.betiuk@imp.lukasiewicz.gov.pl

**Keywords:** Recatest, Recalo, CrN/CrCN, AlCrN, Alcrona, Balinit, Calotest, PVD, CVD

## Abstract

The purpose of the paper is to present a new Recatest testing technique which uses a series of abrasions within a scratch and its innovative application to describe selected quantitative parameters of locally, plastically deformed substrate and coating materials detected on the spherical microsection in the scratch test. The exposed material structures are subject to a metallographic analysis which allows for the determination of the quantitative parameters, which in turn allow for a description of the change in dynamics of the coating structure within the scratch area as a function of load. These parameters include scratch depth (*h_s_*), coating thickness (*h*_1_), flash height (*h_oc_*, *h_os_*), depth of intended material (*h_d_*), material depth under scratch (*h_cp_*), and material depth under coating (*h_db_*). The paper also includes a description of the Recalo test device designed by the authors, which is used to make a series of spherical abrasion traces on the scratch surface. Recalo is dedicated to the Recatest technique. The analysed material was the CrN/CrCN/HS6-5-2, AlCrN -Alcrona-Balinit/D2 coatings deposited on tool steels.

## 1. Introduction

Modern diffusion layers and coatings are multifunctional materials. They have a complex phase structure and are multilayered. The coatings and layers with the substrate form an aerological system. The term “aerological system” (burk) relates to an arrangement of surface layers of modified substrate (core) which is constituted with various technologies and changes during use. The system will be formed as a result of a complex material structure being formed on the substrate surface, including a layered composition of materials of varying physical, chemical and mechanical parameters. The CVD (chemical vapor deposition), PVD (physical vapor deposition) coatings form an aerological system adapted to the operational conditions of use. The improvement of such system based on the market requirements is a stimulus of progress in the field of material technology and complex structure materials.

The thickness of coatings obtained with the use of galvanic methods and CVD, PA CVD (chemical vapor deposition with plasma activation), PVD, PA PVD (physical vapor deposition with plasma activation) is 0.2–30 µm. Most coating materials, such as carbides, nitrides, carbon coatings, e.g., TiN; TiCN; TiC [1,2], CrN [3], CrN/CrCN [4,5,6], AlTiN; TiAlCrN [7], ArCrN; TiAlN [8], and DLC [5,9], on tools and machine parts have a thickness within the dimensional tolerance required by the tool geometry. The thickness of such functional coatings is within the range of 0.2–5 µm. The development of material engineering of tool and machine coatings is based on the structural and phase synergism or antagonism of primary structures in order to obtain totally new operational properties, such as better hardness, lower friction coefficient, improved strength, hydrophobicity, etc. [7,10,11,12]. The improvement of coating technologies is inextricably connected with the search for investigation tools for quick evaluation of the tool quality and structural construction revealed on a metallographic microsection. One of the basic quality acceptance criteria of coatings is thickness measurement, evaluation of the metallographic structure, including the areas neighbouring the substrate–adhesion to the substrate [13,14,15]. 

In traditional metallographic examination, the layer thickness and structure are evaluated based on optical observation and measurements of perpendicular polished sections. It is very difficult to identify the structure of thin layers (0.1–5 µm) with an optical microscope. The oblique polished section (angle 2–5°) is convenient as it physically enlarges the analysed area, but is labour-intensive (precise fastening or inclusion of specimens in holders in order to eliminate the edge rounding effect during grinding and polishing), and consequently is not widely used. 

The spherical microsection method with a large-diameter ball is easier and performs better during thickness measurements, and particularly during the analysis of thin multilayer coatings [13,16]. The method is quick and simple and is defined in the ISO 26423: 2016 standard [17]. The metallographic microsection made by a ball has the features of an oblique microsection, physically extending the area of the analysed layer. A slight microsection sphericity does not affect the quality of the microscopic examination.

## 2. Methods and Materials

### 2.1. Spherical Microsections of PVD Coatings-Recalo

**Figure 1:**
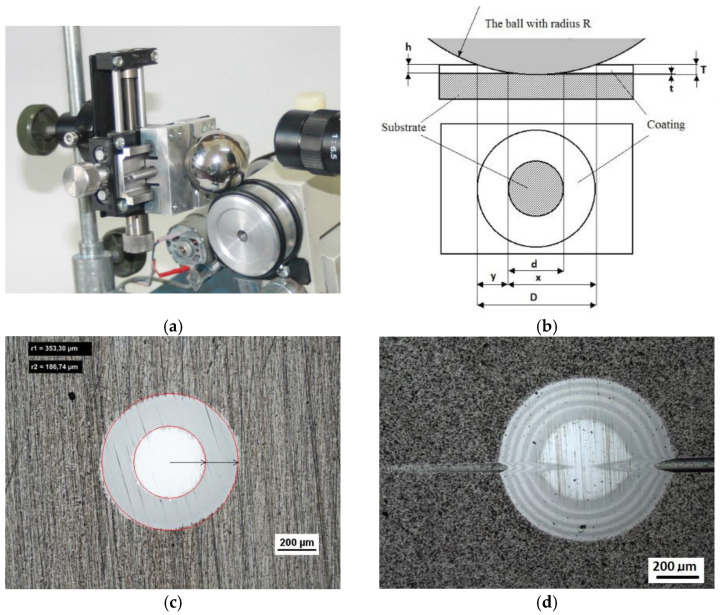
Recalo; (**a**)—a ball in a tribological node, (**b**)—a scheme of the geometry of the spherical microsection with an indication of measurement parameters for determining thickness of the coating, (**c**)—a image of multi-layer PVD CrN (Nikon), (**d**)—an image of multi-layer PVD coating spherical microsections CrN/CrCN ×4 /HS6-5-2 (Nikon). All units appearing in drawings are measurement in (µm).

Recalo and Recatests, the dedicated examination techniques, allow the structures to be revealed on the spherical microsection with the employment of optical, scanning, and laser microscopy, Figure 1. It allows the following identifications to be made:

- thickness measurement of the coating and of the coating layers,
- evaluation of the coating and layer structure in metallographic examinations,
- microhardness examination on the developed coating cross section,
- distribution of chemical elements on the developed coating cross section,
- quality evaluation of mutual adhesion of the coating layers,
- quality evaluation of adhesion of the coating to the substrate,
- quantitative surface roughness measurement,
- preliminary evaluation of local friction wear,
- density of coating materials (defined abrasion cap geometry),
- point marking of surface with microabrasion, and
- formation of reactive environment diffusion channels to the coating and substrate zones.

**Figure 2:**
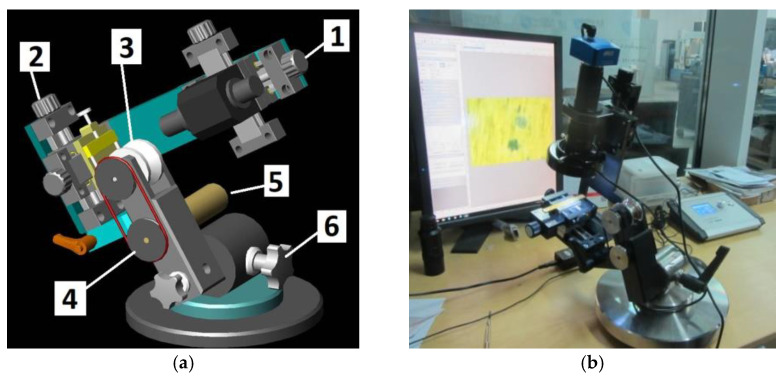
A new type of Recalo (**a**)—an assembly drawing structure; 1—a optical head with sight, y-x axis sample; 2—a head with micro vice; 3—a ball drive roller; 4—a belt transmission; 5—a x-y axis electric motor, rotation speed 100 ÷ 350 n/min; 6—a angular head with movement lock, (**b**)—a version dedicated to the Recatest research technology with precise location of the spherical microsection.

The diagram of a spherical microsection revealing the coating structure with characteristic geometric parameters measured on the microsection is shown in Figure 1b. Recalo was modernised in order to increase its precision, improve the measurement reliability, adapt it for cooperation with a PC (image analysis) (version 3.2, 2020, NIS Elements BR, Tokyo, Japan), and enhance the design ergonomics, as presented in Figure 2.

**Figure 3:**
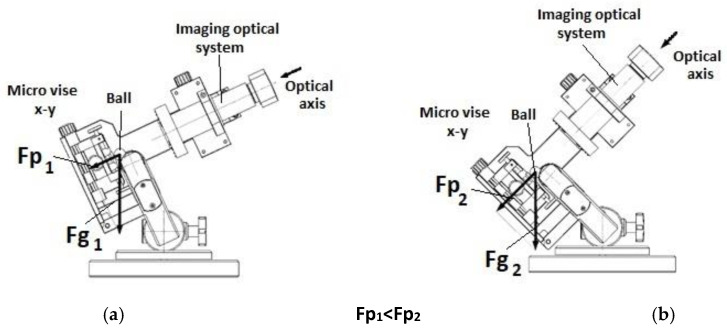
Recalo stand layout, friction node positioning (**a**)—measurement head position at low friction node load; (**b**)—measurement head position at high friction node load.

In the light of deficiencies of generally used scratch test methods and Calotest which involve obtaining incomplete qualitative and quantitative data on coating and substrate materials, the authors have decided to design a device called Recalo. The Recalo brings new testing opportunities in the area of precise metallographic evaluation. It allows making a spherical microsection in a dedicated point of the surface, e.g., in the scratch area. It is worth emphasizing that numerous abrasions can be made along the scratch (3–8 abrasions). The precision of making the microsections has become a source of new qualitative and quantitative parameters concerning the locally deformed structures. It is also worth mentioning that the Recalo also allows the evaluation of structures under the scratch surface and on the hardness indentations rim. We have pioneered this technique in Poland. The latest Recalo version was designed and made at Biuro Konstrukcyjno–Wdrożeniowe Piotr Domanowski of Bydgoszcz, Poland. For the latest testing system (Version KT–1, Year 2019, Biuro Konstrukcyjno–Wdrożeniowe Piotr Domanowski, Bydgoszcz, Poland), see Figure 2 and Figure 3.

The innovative elements included:

- a new specialized optical system integrated with Recalo for using digital cameras,
- a bench (micro-vice) integrated with the x-y cross table for specimen positioning,
- a rotary body ensuring better access,
- an optical system precisely positioned in x–y or x–y–z axes,
- a microprocessor controller with a display of set and current operating parameters,
- a digital camera with a dedicated lens, magnifying software for analysing images, including spherical microsections.

**Figure 4:**
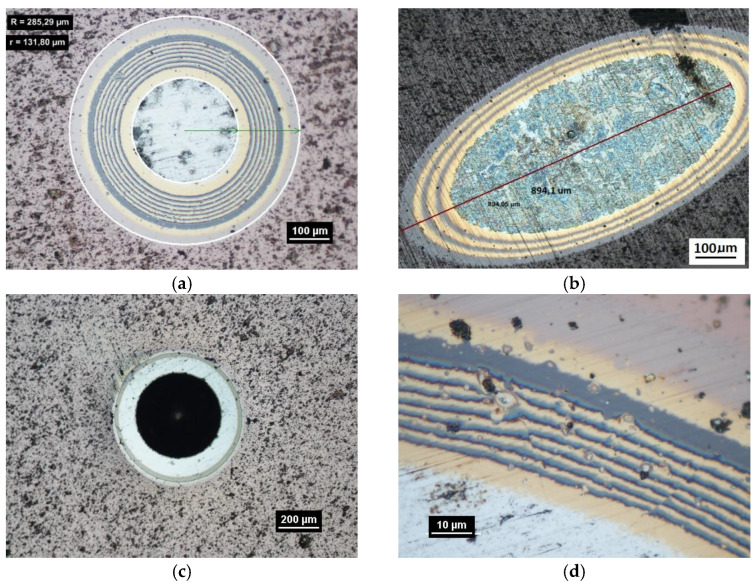
The structure of the multilayer coatings TiN/TiAlN/TiCN/ HS6-5-2 on the flat surface, (**a**)—a polished surface, (**b**)—a structure of the multilayer coatings TiN/TiAlN/ HS6-5-2 on the cylindrical cutter, (**c**)—a precise spherical polished surface in the center axis of the Rockwell test, the coating adhesion (**d**)—a multilayer coatings TiN/TiAlN/TiCN/ HS6-5-2 of the Rockwell test, the coating adhesion.

The making of a spherical microsections in a precisely defined specimen point, e.g., in the scratch area, HRC indentations, (Figure 4c,d) is related to other activities, such as:

- fastening the specimen in the micro-vice clamps with x-y positioning relative to the microscope and image recorder optical axis,
- surface visual inspection through the microscope or at the computer screen (optical system with a CCD camera)(Delta Optical, Mińsk Mazowiecki, Poland) and choosing the test location Figure 1,
- optical calibration of the image (sharpness, magnification) along with determination of the observation optical axis which changes during the test. The optical calibration involves the first, blind abrasion test with a ball of minimum depth outside the test area. The resultant micro-abrasion is a point at which the optical axis of the image recording system is trained,
- making of a spherical microsection of a determined diameter in the determined place requires that this place be located in the optical axis. To this purpose, the test place is moved in x–y positions along the optical axis.

The Recalo test stand design allows a stepless adjustment of the free-rolling abrasion ball without changing the position of support points on the roll and the tested surface. The angle changes can be made during the friction process with the ball (Figure 3). It is particularly important when making precise, shallow microsections on brittle surfaces, e.g., Si specimens with PVD coatings. The stability of the image system optical axis allows controlling the process of ball indentation and exposing the deeper structures. The range is controlled by periodic removal of the ball from the roll and exposing the field of operation. After the observation, the ball is replaced in the friction nodes. 

**Figure 5:**
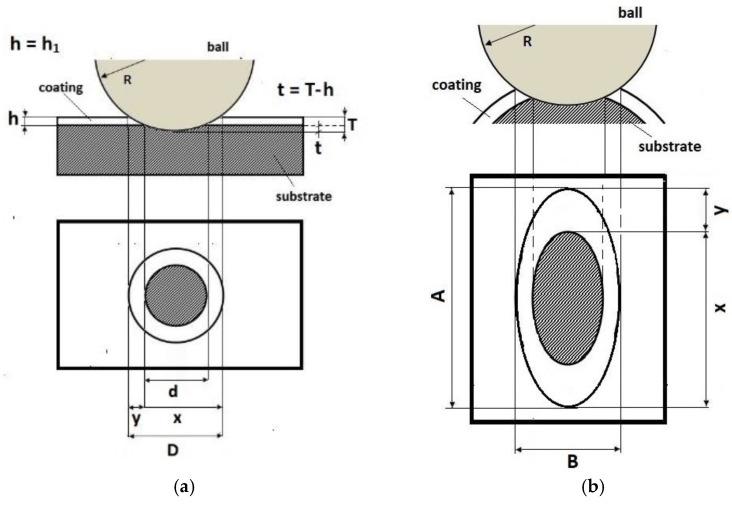

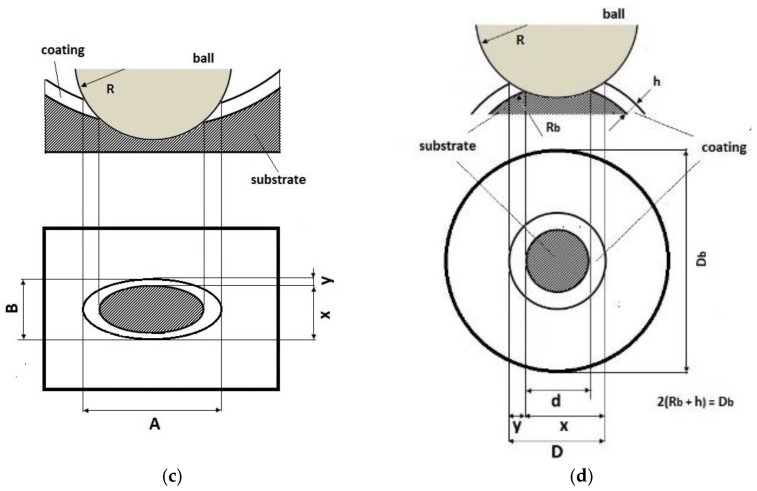
A scheme of the geometry of the spherical microsection with an indication of measurement parameters for determining thickness of the mono-coating, (**a**)—a flat surface, (**b**)—a convex cylindrical surface, (**c**)—a concave cylindrical surface, (**d**)—a spherical surface. A—major axis of the ellipse, B—minor axis of the ellipse, R—ball radius, D—diameter spherical cap of the coating and substrate, d—diameter spherical cap of the substrate, dimensions x and y the thickness of the coating h is calculated by h = xy/2R, h—thickness coatings, Db—diameter of the ball with the coating, Rb—radius of the ball before coating.

**Figure 6:**
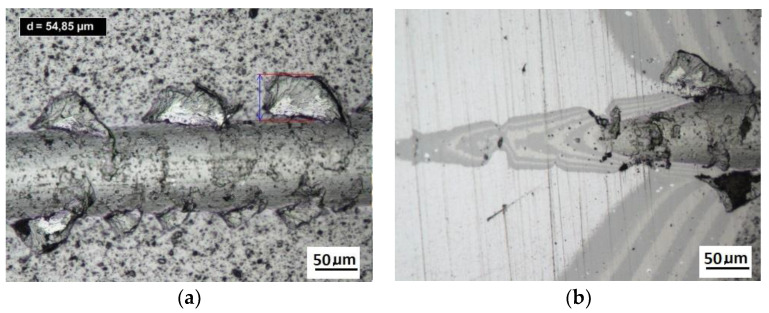
The structure of the multilayer coatings 4× (CrCN/CrN)/HS6-5-2 on the cylindrical cutter, (**a**)—a deformed coating in the scratch area, cohesive chipping, (**b**)—a deformed coating on the spherical microsection.

**Figure 7:**
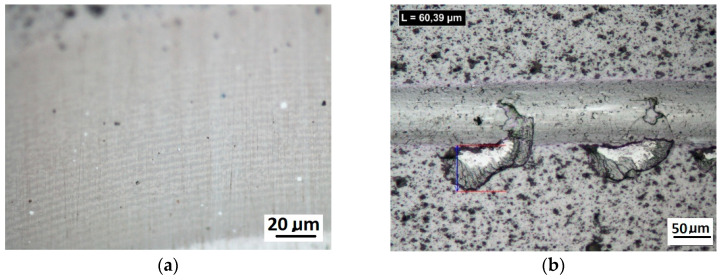

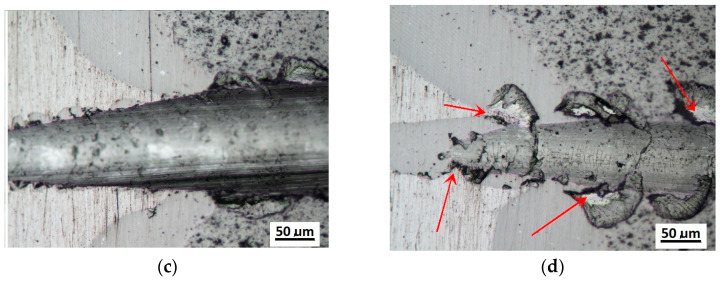
The structure of the multilayer coatings 32×(CrCN/CrN)/HS6-5-2 on the cylindrical cutter, (**a**)—multilayer coatings 32×(CrCN/CrN), (**b**)—a deformed coating in the scratch area, cohesive chipping, (**c**,**d**)—a cohesive chipping in the area of the upper layers of the coating.

The spherical microsection can be made on planar, cylindrical, conical and spherical concave and convex surfaces (Figure 5). With the precisely defined geometry of the microsection surface to be analysed, it is possible to make quantitative measurements of revealed structures, i.e., measurements of their thickness, porosity, micro-porosity and, for multilayer coatings, the structure modulation parameter, propagating cracks, chipping in the structure of the coating (Figure 4, Figure 6 and Figure 7). Using advanced ion etching techniques for surface preparation, it is possible to obtain coatings with high mutual adhesion. To ensure such quality of areological systems it is necessary to carry out a precise and accurate analysis of structures before and after operational tests under the conditions simulating their wear during normal work. The basic property which characterises coatings and layers is their thickness. 

A prerequisite for measuring the thickness on metallographic microsections is a strong optical contrast between examined structures which makes the determination of physical phase boundaries possible. The methods for measuring the coating thickness on surfaces with various geometrical properties are listed in Table 1. 

In Table 1, the following parameters of revealed structure play a role of measured quantities: *R*—radius of the ball used for making the microsection (µm), *R_b_*—radius of the measured ball (µm), *T*—depth of the spherical microsection (µm), *t*—depth penetration in the base material, *h*_1_—vcoating thickness (µm), *d*, *x*, *y*, *D* measurement data defined on the spherical microsection (µm).

### 2.2. Recatest Technique—Qualitative and Quantitative Analysis of the Revealed Structures of Coating Materials

The Recatest technique is a supplement to the scratch test in PVD coating quality tests. Recatest provides more precise evaluation of the metallographic microstructure of the substrate-coating areological system strained during the scratch test [9]. 

One of the quality measures for coatings on machine parts and tools working in heavily loaded tribological pairs is their adhesion to a modified substrate. For evaluation of the adhesion the method of scratch test is widely used. The scratch test method consists in making scratches on the surface of a layer-substrate system with an indenter of a specified shape (usually the diamond indenter type Rockwell C (Rockwell—WILSON, New York, NY, USA). It is possible to perform the scratch test under an increasing or constant load. The most frequently used REVETEST–RST (Anton Par) (CSEM Instruments, Neuchatel, Switzerland) allows one to make the scratch test with a loading force within the range of 0–200 N, at a loading rate of 100 N/min and indenter travel speed of 10 mm/min. The tests are performed in compliance with ASTM C1624 [18], ISO 20502:2005 [19]. 

Damage of the layer-substrate system is detected and evaluated based on a direct microscopic observation of the scratch and on a measurement of acoustic emission and tangential force. The normal force at which the damage appears is referred to as a critical load (Lc). The main cases of changes and damage occurring during the scratch test can be classified as plastic deformation, cracking (Lc1), peeling (chipping) (Lc2), and penetration of the coating into the substrate in the central part of the scratch trace (Lc3). Information on the phenomena of degradation of the coating structures coming only from the scratch surface is incomplete. The cracks and edges visible on the surface of the cracks, cohesive chipping and delamination only inform about the occurrence of such phenomena in correlation with the force loading the areological system. Analysis of the image of the degraded structure on the spherical microsection surface (Figure 6 and Figure 7) allows for a precise evaluation of the mechanical degradation of the internal structures of multilayer coatings. Crack scattering phenomena at the phase boundaries of layers, separations, inclusions, areas of selective delamination, etc. become visible.

**Figure 8:**
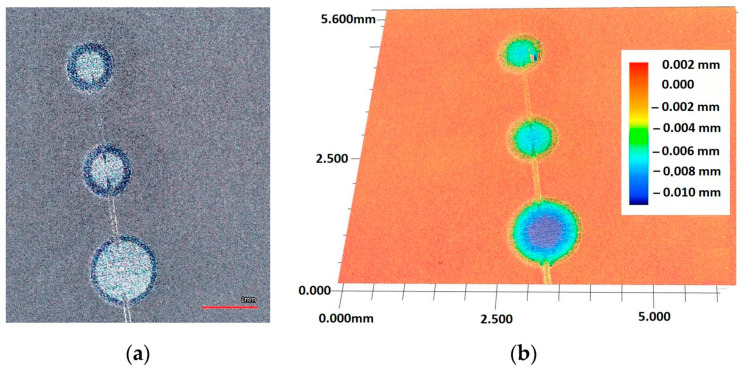
The spherical microsection in area scratches, (**a**)—a three tracks spherical micros section in area scratches, (**b**)—a map of surface microgeometry changes (Keyence VR-5000).

The Recatest principle involves a combination of two known coating testing techniques: the scratch test with a diamond indenter RST (CSEM Instruments, Neuchatel, Switzerland) and a precisely localised spherical microsection with the use of Recalo (proprietary patented test rig). The Recatest technique involves making a localised spherical microsection in the area where scratches were previously made on the areological system being examined (Figure 8). 

The innovativeness of the Recatest testing technique lies in making a few microsections along the scratch (3–8 microsections). As a result, quantitative and qualitative information is obtained on the structure change dynamics in the deformed coated material as a function of the increasing load of the indenter which generates a scratch both on the material surface and its skew profiles.

The precisely defined place and geometry of analysed microstructure areas on the spherical microsection are a source of new qualitative and quantitative data that evaluate the system mechanical durability (cracking, chipping, delamination). In industrial and laboratory practice, the normalised basic scratch test is carried out using a diamond indenter in an automatic cycle with a controlled penetrator pressure force and load speed, while recording acoustic emission.

**Figure 9:**
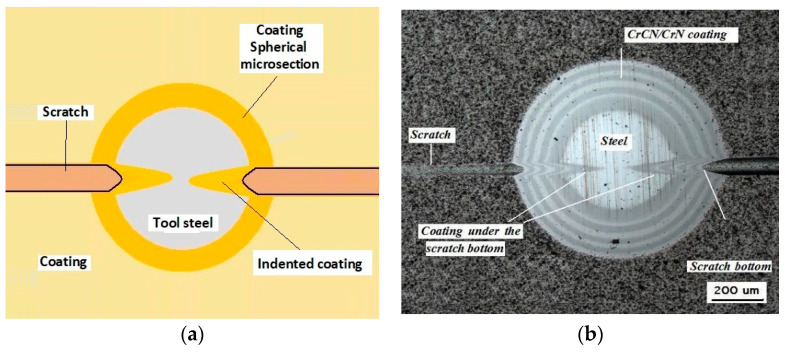
Diagrams and a microstructure image (spherical microsection) of the coating areological system in the scratch area: (**a**)—a spherical microsection diagram, (**b**)—microstructure of the CrN coating revealed on the spherical microsection with a description (30 mm ball).

**Figure 10:**
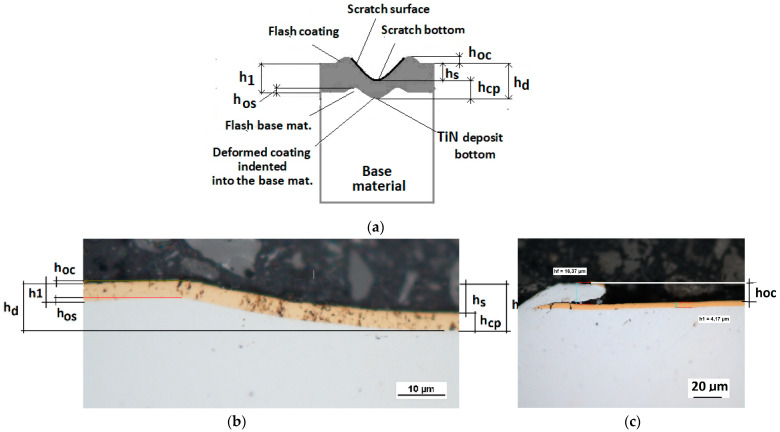
Diagrams and a microstructure image (perpendicular microsection ) of the coating areological system in the scratch area: (**a**)—a spherical microsection diagram, (**b**)—a microstructure of the TiN coating a perpendicular microsection diagram, letters are assigned to the measured structure: *h*_1_—coating thickness, *h_d_*—scratch depth, *h_oc_*—flash height, *h_d_*—coating indentation depth, *h_s_*—scratch bottom depth, *h_cp_*—thickness coating pressing under the scratch bottom, *h_os_*—height of the outflow of the base material, (**c**)—a microstructure of the TiN coating a perpendicular microsection diagram, *h_oc_*—height of the outflow of the coating material parameter.

The Recatest method involves making a spherical microsection on the rig and Recalo ensures:

- precise revealing of the microstructure of the deformed coating,
- a quantitative analysis of changes of the microstructure geometry parameters in the deformed coating under the bottom of the scratch,
- a graphical illustration of microstructure geometry changes within the scratch in correlation with scratch test parameters–critical forces Lc1, Lc2, Lc3,
- An example of the diagram and structure of the deformed CrN, TiN coating revealed on the spherical microsection within the scratch area and identified quantitative parameters are shown in Figure 9 and Figure 10.

### 2.3. The Analysis of the Coating Structure on the Spherical Microsection within the Area of Scratches

**Figure 11:**
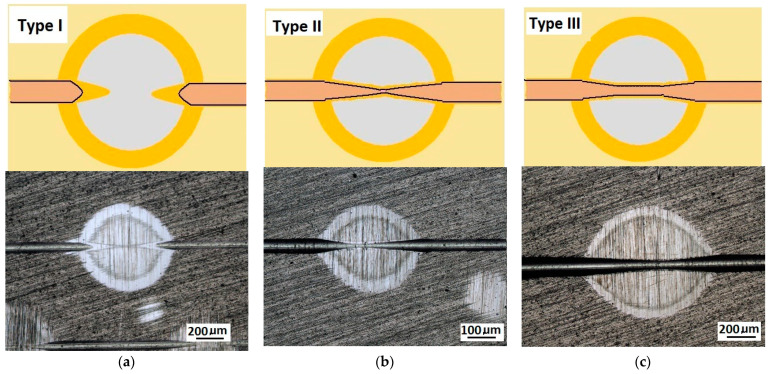
Diagrams and a microstructure image of the coating areological system in the scratch area: (**a**)—a spherical microsection diagram the microstructure CrN type I, (**b**)—a spherical microsection diagram the microstructure CrN type II, (**c**)—a spherical microsection diagram the microstructure CrN type III.

While conducting optical observations of the spherical microsection in the area of the scratch, three basic types of structural images can be defined. The differentiation results from the mutual relation of the depth of the scratch bottom and the depth of the spherical microsection (Figure 11). So, three types of structures can be defined in which the spherical microsection is:

- Deeper than the scratched bottom: type I.
- At the border of the scratching: type II.
- Less deep than the scratched bottom: type III.

**Figure 12:**
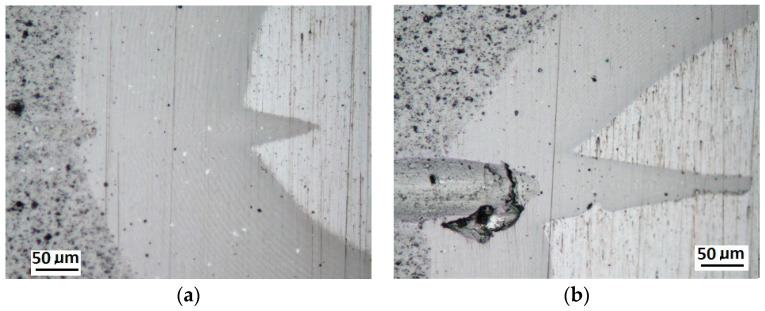
Diagrams and a microstructure image of the coating areological system in the scratch area: (**a**)—a spherical microsection diagram, (**b**)—a microstructure of the CrN coating revealed on the spherical microsection with a description (30 mm ball).

The quality of the coating structure change due to the local plastic deformation in the crack test is best performed on the surface of the smooth type I (Figure 11a). This sample shows the crack and its penetration area in the coating material. It is also possible to observe the deformed structure of the coating pressed into the base material. The figure shows the structure of type I of the 16× CrN/CrCN tarpaulin in areas that differ in the depth of the crack, the amount of load on the head. The increase in the impact force of the indenter causes the phenomenon of brittle cracking of the coating and an increase in the depth of its insertion. The scratch coating material does not degrade much (Figure 12b).

**Figure 13:**
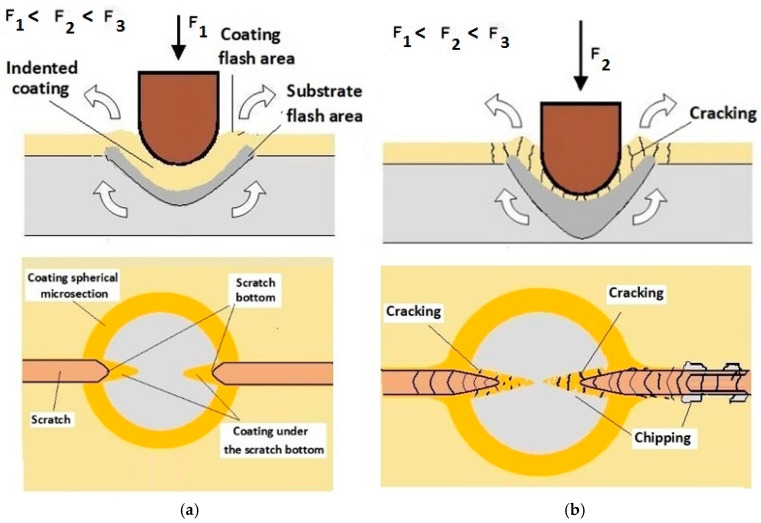

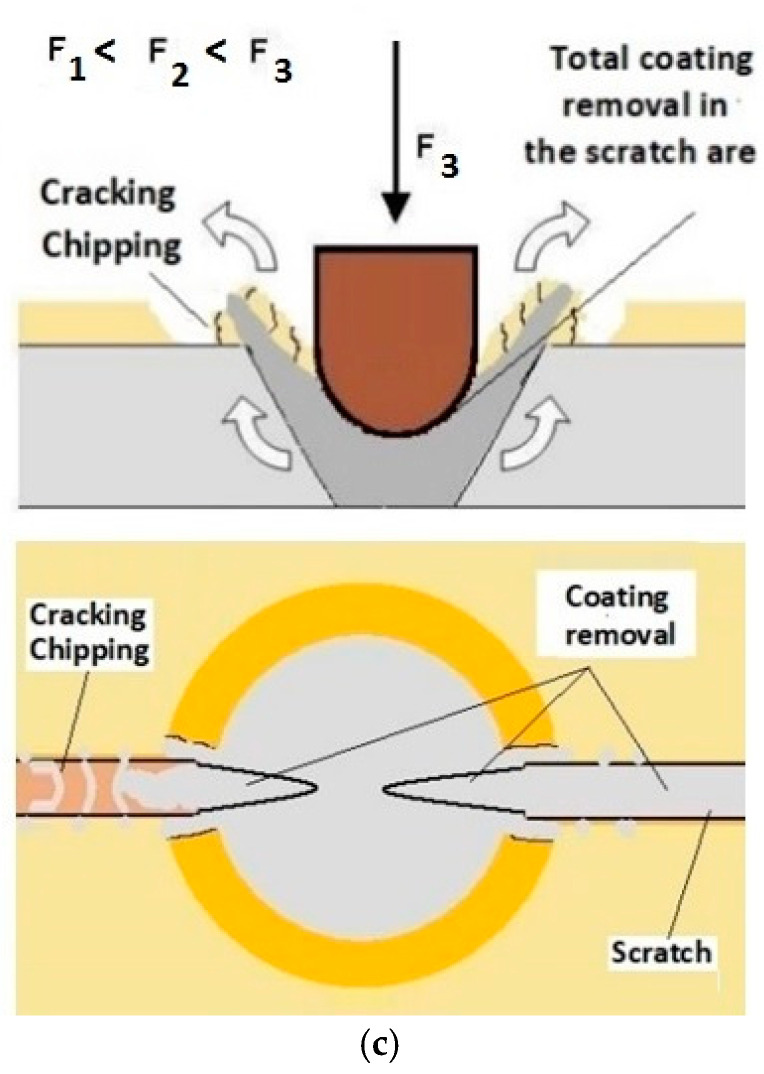
This is a figure. Schemes follow the same formatting; (**a**) a type A microstructure, the coating microstructure with structural continuity under the scratch bottom; (**b**) a type B microstructure, the coating microstructure with partial loss of structural continuity under the scratch bottom; (**c**) a type C microstructure, the coating microstructure with total loss of structural continuity under the scratch.

Schemes follow the same formatting, this is a Figure 13, the caste A, B, C:

- Case A represents a situation when a continuous uniform coating microstructure is maintained under the scratch bottom and on the scratch rim within the local deformation force Fn (N). The system quality is high. The basic quantitative parameter characterising this case is the maximum coating indentation depth under the scratch bottom *h_cp_* [µm] and the resulting depth of indented coating *h_d_* (µm),
- Case B shows a coating microstructure under the scratch bottom when the local deformation force Fn (N) is exceeded. The coating undergoes deformation in the form of cracking, chipping and delamination. The coating microstructure partially loses its continuity. The system quality is still satisfactory,
- Case C corresponds to the total coating removal from the plastic strain area when the local deformation force Fn (N) is exceeded. The microstructure observation on the spherical microsection does not indicate the presence of the coating material under the scratch bottom and on the scratch rim. The system quality is poor.

**Figure 14:**
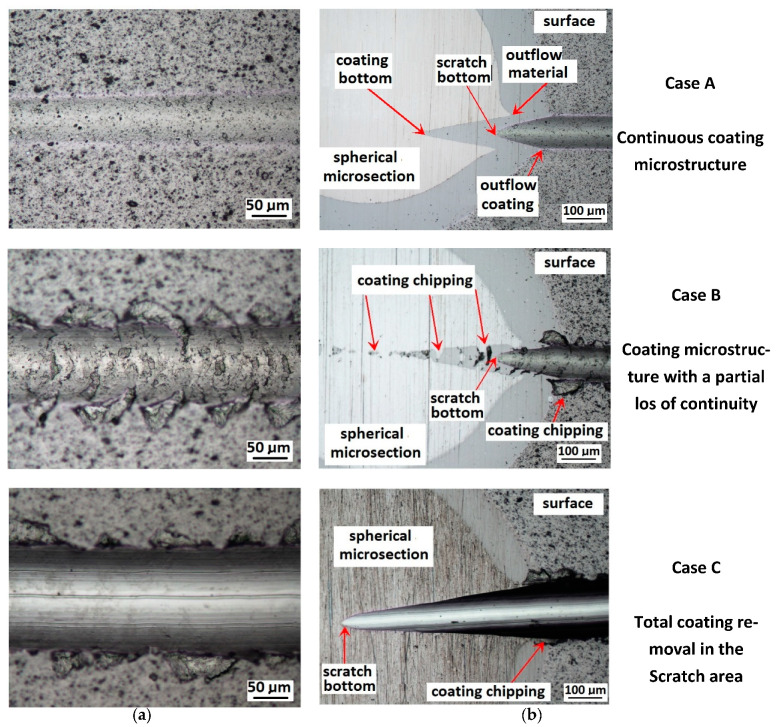
A surface structure in Recatest. Microstructure images; (**a**)—a deformed coating in the scratch area, (**b**)—a deformed coating on the spherical microsection.

Figure 14 shows the surface structure in areas characteristic for a CrN coating in Recatest testing for the three cases described above. 

#### Recatest Analysis of Case A Coating Structures

For analysing the PVD coating strained during scratch test and revealed using the Recatest technique, the coordinates of structure characteristic points must be measured very precisely. Most often, these coordinates are determined by the measure of concentric radius originated in the centre of the rubbed out area (Figure 13).

**Figure 15:**
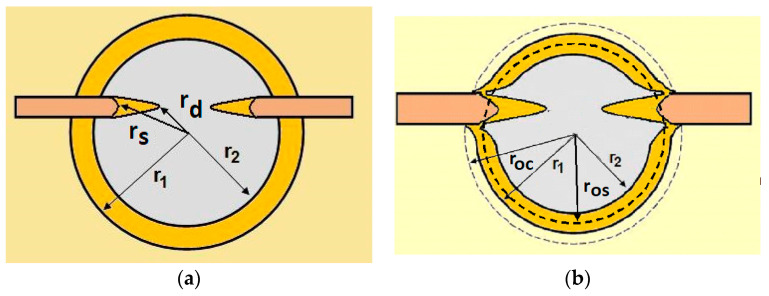
The PVD coatings and A type traces of the scratch revealed on the surface of the spherical cut on steel; (**a**)—a deformed coating without flashing on the edge of the crack, (**b**)—a deformed coating with a flash on the edge of the crack, the outflow of the coating material, the base material. Explanation of the markings; rd—radius of the depth of pressing the deposited coating, rs—radius to the apex of the scratch bottom, r1—radius of the base of de cap, r2—radius of the lower limit of coating, ros—radius of the upper edge of the outflow substrate material, roc—radius of the upper edge of the outflow coating material.

The radii specified in Figure 14 correspond to the respective distances between the centre of the spherical microsection and a given characteristic point of the coating structure, namely: r_s_ determines the scratch bottom point, r_d_ determines the distance to the coating end point under the scratch, r_oc_ determines the radius of the outflow coating material, r_os_ determines the radius of the outflow base material, r_1_ determines the radius of the bowl boundary, and r_2_ indicates the radius on the coating end boundary under the surface. Based on measurement data from Figure 14 and Figure 15 and mathematical relationships between geometrical points on the spherical surface, the following values can be determined: coating thickness, scratch bottom depth, and thickness of the coating underneath the scratch bottom. The geometrical relationships and exemplary calculations conducted on the basis of data from Figure 8 and Figure 9 are shown in Table 2 below.

Additional symbols used in Table 2 have the following meanings: *T*—depth of the spherical microsection; *h*_1_—coating thickness, *h_d_*—scratch depth, *h_oc_*—flash height, *h_db_*—coating indentation depth, *h_s_*—scratch bottom depth, *h_cp_*—thickness coating pressing under the scratch bottom, *h_os_*—height of the outflow of the base material, *R*—ball radius; *D*—diameter of the spherical rubbed out area; *x*, *y*, *d*—measurement data (Table 1).

### 2.4. Experimental Part

In order to illustrate the methodology, we present examples of tests performed on the CrN/CrCN, AlCrN-Alcrona Balinit coatings on tool steels obtained using the PVD Arc (Physical Vapor Deposit an electric Arc) technologies. The results of CrN/CrCN coating tests were published in firmaments in papers [16].

- Testing techniques used

In order to identify the structure CrN/CrCN, Alcrona Balinit^®^ coating (Oerlikon Balzers Coatings, Polkowice, Poland) the following tests: metallographic, scratch test, spherical metallographic section in the area of scratches (Recatest).

- Metallographic tests

For metallographic tests performed on spherical metallographic sections made with a 30-mm diameter steel ball (a steel ball test bed in accordance with the norm ISO 20502:2005 [19]) a Recalo. For surface structures, metallographic images, and geometric measurements, a Nikon LV150 (Nikon, Tokyo, Japan) optical microscope was used. A KYENCE VHS 5000 co-focal microscope (Kyence, Japan Corporation, Osaka, Japan) was also used in the structure tests. The Alcrona coating structure was also shown using the classic technique on the surface of the microsection perpendicular to the substrate using a metallographic test kit from Struers-LoboPol-5 (Struers, Krakow, Poland), 21.

- Scratch tests

Adhesion tests and determination of symptoms of mechanical damage were carried out by the scratching method (the test described by the norm ISO 20502:2005 [19]) on a Revetest^®^ Scratch (CSEM Instruments, Neuchatel, Switzerland) installation, with an increasing load force of 0–100N, 0–150 N and an indenter speed of 10 mm/min. The scratch was made using a Rockwell indenter of a 200 µm vertex radius.

- Recatest

Metallographic spherical microsections were made using a diameter 30 mm steel ball. The microsection location on the scratch area was chosen to include in its space a characteristic symptom of disturbing the coating cohesiveness specified by parameters Lc1, Lc2, Lc3, classically defined. The test point localisation was made in accordance with the procedure described in that paper. 5, 6 spherical microsections were made on the scratch surfaces, trying to obtain the I or II type structure of deformed coatings. (Figure 11). The structure type control was obtained by a sequential interruption of the rubbing process with a ball connected with the optical examination of the friction nide surface. The ball rotation speed was set at 250 n/min. The diamond paste of granulation < 1 µm was used in the friction node in order to accelerate the process of making the spherical microsection. The Recalo head inclination angle was controlled during the test in order to maintain the flexible ball movements. The Recalo head inclination angle range was set at 5°–40°. Using a diameter 30 mm ball (100Cr6 steel) allowed its pressure force on the specimen support point to be adjusted in the F_d_ = 0.2 N–0.7 N range.

### 2.5. Tests of the CrAlN–Alcrona Balinit Coating

**Figure 16:**
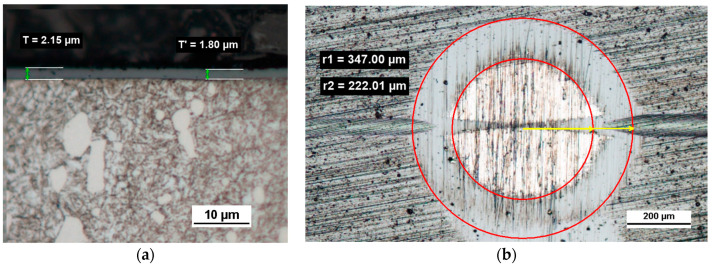
Microstructure images; (**a**)—a metallographic section transverse metallographic, (**b**)—a spherical microsection, yellow arrows, coating thickness measuring radii r1, r2.

The comprehensive tests of PVD Alcrona Balinit coating were performed on a commercial coating on the flat substrate of the D2 steel thermally treated to the hardness of 63 HRC. The revealed coating structure on the transverse and spherical microsection surface is presented in Figure 16. The coating thickness is 2 µm ± 0.2.

**Figure 17:**
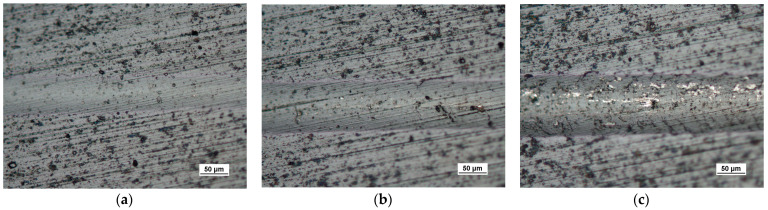
The scratch test, structure of the scratch bottom surface; (**a**)—a load force range for 16–27 N, (**b**)—a load force range for 39–50 N, (**c**)—a load force range for 64–78 N, Fn = 64 N (Lc1).

**Figure 18:**
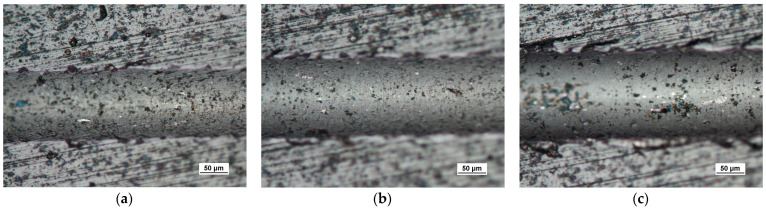
The scratch test, structure of the scratch bottom surface (**a**)—a load force range for 83–99 N Fn = 83 N (Lc2), (**b**)—a load force range for 106–124 N, (**c**)—a load force range for 129–148 N, Fn = 148 N (Lc3).

Scratch tests in the Fn = 0–150 N load range were performed on the coating surface. The scratch length was 11 mm. The visual inspection of the scratch bottom and sides (Figure 16, Figure 17 and Figure 18) allowed determining two types of damage disturbing its cohesion: Lc1 first crack (Figure 17c), Lc2 first chipping on the flash side (Figure 18a). The coating maintained cohesion with the substrate along its entire length, and no adhesion loss was observed. 

**Figure 19:**
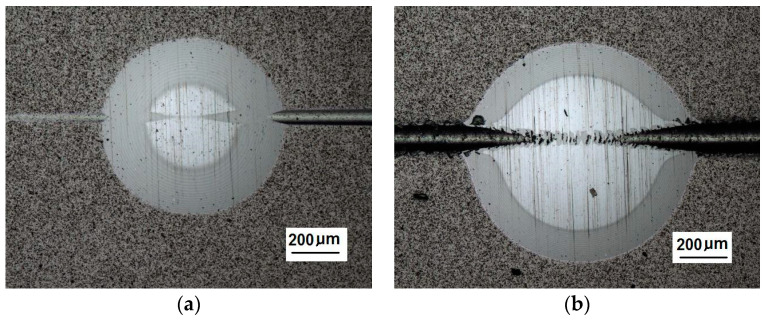
A surface structure in Recatest. Microstructure images; (**a**)—a spherical microsection of the CrCN/CrN/HS6-5-2 coating for 40–46 N (Lc1), (**b**)—a spherical microsection of the CrCN/CrN/HS6-5-2 coating for Fn = 61 N (Lc2).

**Figure 20:**
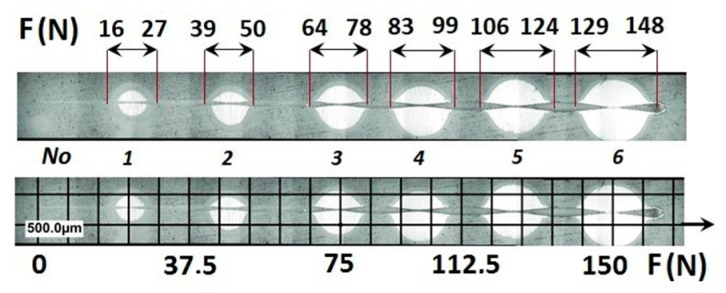
A surface structure in Recatest, a microstructure images spherical microsection of the AlCrN- Balinit Arcrona in six areas of the scratch the spherical microsection, load force range for 1⟶16–27 N; 2⟶39–50 N; 3⟶64–78; 4⟶83–99; 5⟶106–124; 6⟶129–148; No 1–6—Spherical microsection number.

**Figure 21:**
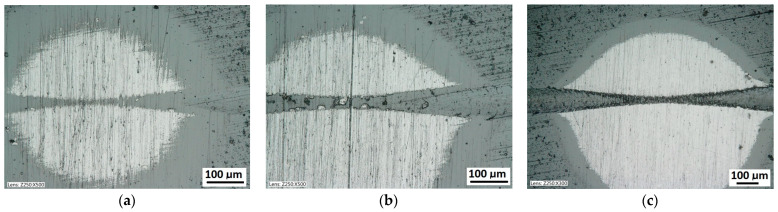
A surface structure in Recatest. Microstructure images spherical microsection of the AlCrN-Alcrona Balinit coating; (**a**)—a load force range for 16–27 N, (**b**)—a load force range for 39–50 N Fn = 50 N (Lc1), (**c**)—a load force range for 64–78 N.

**Figure 22:**
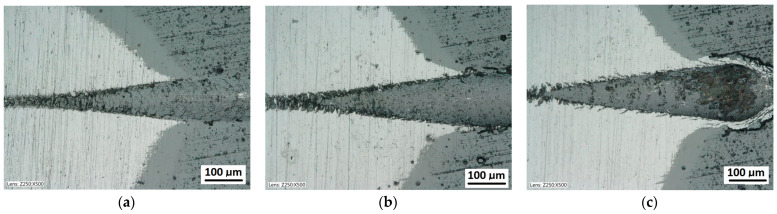
A surface structure in Recatest. Microstructure images spherical microsection of the AlCrN- Balinit Arcrona coating; (**a**)—a load force range for 83–99 N (Lc2), (**b**)—a load force range for 106–24 N, (**c**)—a load force range for 129–148 N (Lc3).

**Figure 23:**
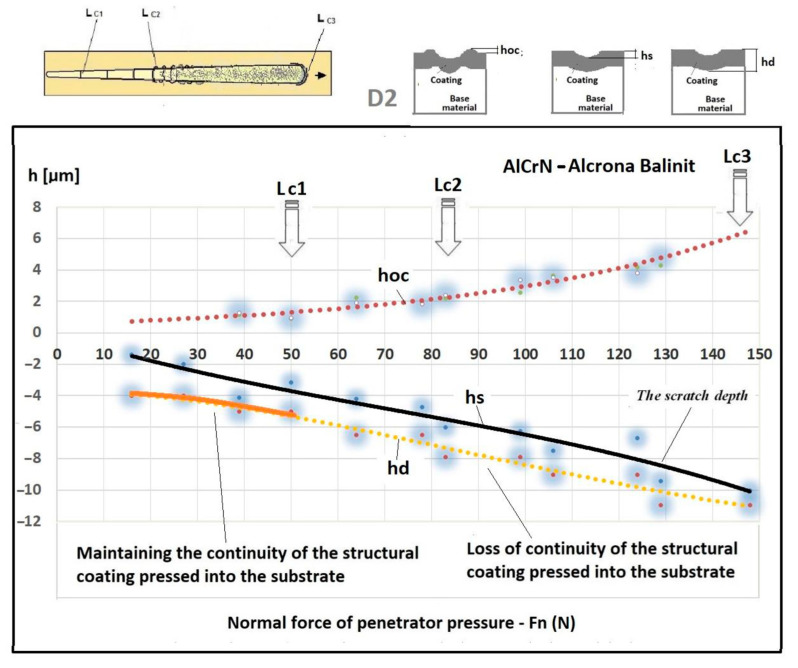
A change of scratch depth, indentation depth and flash height in the scratch area on the AlCrN—Alcrona Balinit coating as a function: *h_s_*(Fn), *h_oc_*(Fn), *h_os_*(Fn) of a normal penetrator loading force Fn in the range from 0 to 150 N, and the structure of the deformed areological system, *h_s_*—scratch bottom depth, *h_cp_*—thickness coating pressing under the scratch bottom, *h_os_*—height of the outflow of the base material.

**Figure 24:**
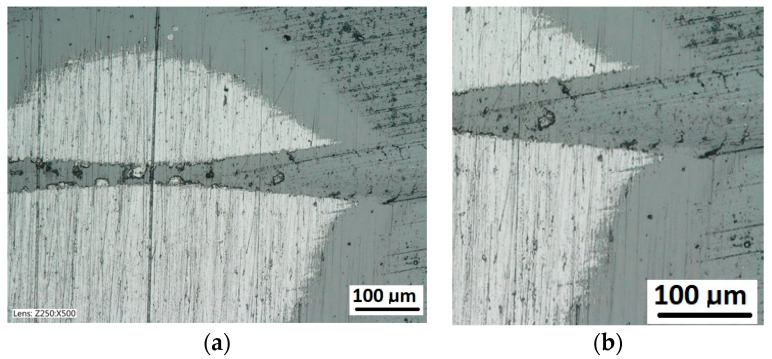
A surface structure in Recatest. Microstructure images; (**a**)—a spherical microsection of the AlCrN-Alcrona Balinit coating for 39–50 N, (**b**)—a spherical microsection of the AlCrN—Alcrona Balinit coating for 50–53 N, Fn = 50 N (Lc1).

A part of the structure test areas wad determined on the scratch length in the coating for the testing with the Recatest method. The microsection place on the scratch surface was localised in the image in an optical system with internal sights. The position of the tested specimen and the test field was determined by means of an angle of the Recalo head (Figure 3) and the rubbing time of a dia 30 mm ball with diamond paste was determined experimentally. The form of the exposed coating structure was periodically checked during the making of the spherical abrasion. The structure check involved the ball lifting and cleaning of the friction nodes, visual inspection of the exposed structures. The rubbing with the ball was completed when the structure II type was achieved (Figure 11b and Figure 19a). In order to partially improve the image quality of the exposed surface after the rubbing node cleaning, an additional rubbing with a clean ball was performed for maximum 30 s. this activity was repeated several times. The images of 6 abrasions on the scratch surface with marked indenter pressure force changes are presented in Figure 20. The analysis of the image of the exposed II type structure coating and the scratch surface allow a qualitative evaluation of the coating material behaviour in boundary areas not identified in a traditional scratch test methodology. These areas include the coating structures under the scratch bottom (coating pressed into the substrate material) (Figure 21 and Figure 22), coating, and the substrate material structure in the upper scratch area, the so-called flashes (Figure 23 and Figure 24). 

An important feature and advantage of the Recatest technique is the possibility of achieving additional quantitative parameters characterising the tested areological system: *h_s_*, *h_oc_*, *h_os_*. Being able to make several spherical microsections in the scratch area, it is possible to assess the change dynamics of the aforementioned parameters as a function of the indenter pressing force. The determined parameters included: scratch bottom depth, substrate and coating material flash height, substrate material flash height, depth *T* of coating visual inspection under the scratch bottom.

**Figure 25:**
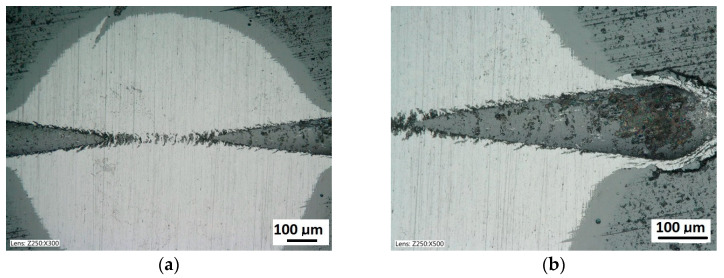
A surface structure in Recatest. Microstructure images; (**a**)—a spherical microsection of the AlCrN-Balinit Arcrona coating for 116–124 N, (**b**)—a spherical microsection of the AlCrN-Balinit Arcrona coating for 140–150 N (Lc3).

The exposed structures are presented in Figure 21 and Figure 22. The analysis of the deformed coating images indicates that the coating maintains cohesion in the entire scratch test range. The identified cracks in the bottom of scratch Lc1 (Figure 21b) appear at 50 N, and adhesion micro-chippings on the flash side Lc2 (Figure 22a) at 84 N are small and have no significant growth dynamics. The adhesion loss of coating Lc3 (Figure 22c) occurs when the pressure force exceeds 148 N and is evident in the area of the most intensive deformation field, that is at the scratch face. The coating adhesion loss covers only the flat area and does not propagate further on the flat surface. A precise visual inspection of the border areas, i.e., the scratch edge and bottom (Figure 17 and Figure 18), scratch edge and bottom exposed on the spherical microsection (Figure 24 and Figure 25), coating pressed into the substrate (Figure 24) and the scratch edge on spherical microsections (Figure 25) indicates that the AlCrN Alcrona Balinit (Balzers, Liechtenstein) coating material does not degrade significantly in the scratch test range. Appearing symptoms of cracking Lc1 and chipping Lc2 are of cohesive character. The deformed coating material is pressed under the scratch bottom (Figure 24), undergoing cohesive fragmentation and flow phenomena along with the substrate material Figure 25. The qualitative description of the AlCrN/D2 aerological system behaviour can be characterised using the structure geometry quantitative data (Table 3) determined with the Recatest technique. The illustrations of the structure change dynamics in the scratch area and of coating behaviour under the scratch bottom are presented in Figure 23. These data (Table 3, Figure 23) indicate that that the size of coating material flashes on the scratch size is even and occur in parallel. This phenomenon, of the significant size of 1 µm (coating thickness 2 µm), can be observed at Fn = 10 N. The scratch side flash size *h_oc_*, at which the Lc3-148 N coating fragmentation occurs is 6 µm, and the scratch depth is *h_s_* = 10 µm. The coating material fragments are pressed under the scratch bottom to the depth of *T* = *h_d_* = 11 µm.

To summarise, it can be said that the Racatests of the AlCrN Alcrona Balinit coating on the quenched and tempered D_2_ steel have shown the high mechanical cohesion of the coating in the scratch area up to Fn = 148 N. The coating does not delaminate in the entire load range, and the coating material is pressed under the scratch bottom and subjected to fragmentation and flow along with the substrate. This phenomenon is observed to the depth of *h_d_* = 11 µm Figure 24 and Figure 25. The statistical measurement error in this method with microsections of diameter up to 2 mm does not exceed 10%. 

## 3. Research Results and Discussion

### Tests of the (CrN/CrCN)×16/ HS6-5-2 Coating

Multilayer CrN/CrCN coatings with modulation 16 (modulation parameter λ [0.3 µm]) were deposited on a substrate of heat toughened HS6-5-2 type tool steel with a hardness of 62HRC in a plasma stream generated in a low-pressure arc plasma source by means of PVD-Arc technology. 

The coating structure was exposed on a spherical microsection, and the identified coating thickness was 4.9 µm ± 0.2. 

**Figure 26:**
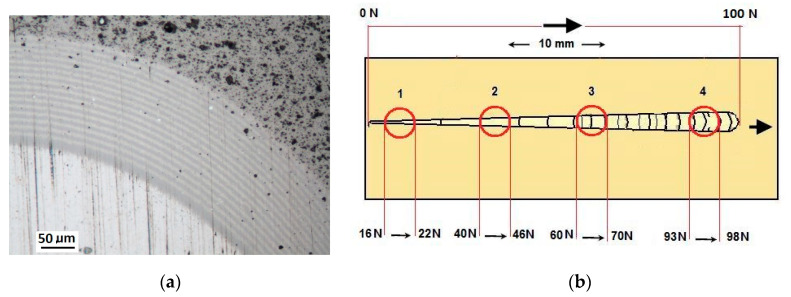
Spherical microsections in the scratch area on the 16× CrCN/CrN coating. A scratch made with a 0–100 N rising load: (**a**)—a spherical microsection of the 16× CrCN/CrN coating; (**b**)—a diagram of the herical microsection location on the scratch area.

**Figure 27:**
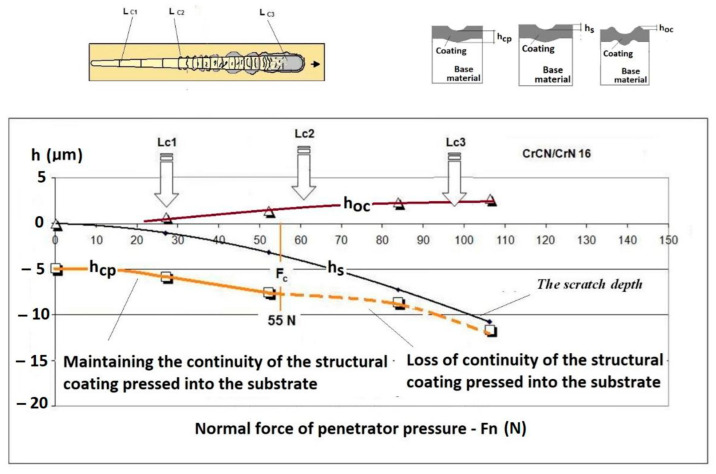
A change of scratch depth, indentation depth and flash height in the scratch area on the PVD coating as a function of a normal penetrator loading force Fn in the range from 0 to 150 N, and the structure of the deformed areological system, *h_s_*—scratch bottom depth, *h_cp_*—thickness coating pressing under the scratch bottom, *h_os_*—height of the outflow of the base material.

With the quantitative data obtained from the analysis of spherical microsections in a few scratch areas it is possible to determine microstructure change dynamics for a coating areological system as a function of a normal penetrator loading force Fn (Figure 26b). Such dynamics refers to the scratch bottom *h_s_*(Fn), system flash height *h_oc_* (Fn), coating indentation depth into the surface h_cp_(Fn). With the analysis of curves h_s_(Fn) and h_cp_(Fn) it is possible to determine the range of penetrator forces which define the microstructural durability of the tested areological system (Figure 19 and Figure 27). In the 16× CrCN/CrN coating system tested, the critical value for the destruction of the coating at the bottom of the crack is plastic deformation, force Fn 55 N. The coating loses its structural continuity under the bottom of the crack at a depth of 8 µm. The approximated scratch depth on the basis of the analysed geometric data at the force point F 55 N is 4 µm.

## 4. Conclusions

The analysis of the AlCrN-Alcrona Balinit coating in the scratch test using the Recatest technique proves that the coating has high resistance to local plastic deformations in comparison with the CrN/CrCN coating. The coatings were deposited with the PVD -Arc. The substrate materials were quenched and tempered HS 6-5-2, D2 steel of 63-62 HRC hardness. The criterion for the adhesion and cohesion loss of the Alcrona collating material within the scratch area is Lc3 = 148 N, and the scratch depth *h_s_* = 8 ± 0.5 µm, flash size *h_oc_* = 4 ± 0.5 µm and trace amounts of the coating material pressed into the coating down to the depth of *h_d_* = 11 µm. The CrN/CrCN undergoes destruction at Lc3 = 98 N, and the estimated material pressing depth is *h_d_* = 11 µm.

The image analyses and determination of critical points of deformed structures are a time-consuming operation. The performed tests prove that the image recognition and analysis techniques can positively be used. The structures have distinct shape and geometrical critical points. The introduction of computer data analysis seems to be necessary for a fuller use of the Recatest technique in case of a large amount of analytical data. 

The combination of the scratch test and spherical microsection techniques provides new methods for the precise quality evaluation of coatings and layers subjected to strong elastic and plastic strains. 

The capability for a quick quality assessment of coatings and layers obtained in thermo-chemical and electroplating processes indicates that the examination methods based on the Recatest technique should be implemented. 

The Recatest technique allows for the analysis of the structural changes of the coatings on a macro and micro scale subjected to scratch tests on spherical microsection surfaces. 

The Recatest technique allows to analyze the changes in the structure of the coatings under the scratch bottom and to determine the critical force of breaking the structural continuity of the coating indented into the substrate material.

## Figures and Tables

**Table 1:** A method of the measurement of the thickness of coatings on the spherical cut.

Geometry of Coated Surface	Coatings Thickness (µm)	Depth Penetration (µm)
Planar (Figure a)—The total penetration depth of the ball in the layer and into the substrate is:	-	$T=R-\sqrt{R^{2}-r_{1}^{2}}$
Planar (Figure a)—The depth penetration in the base material is:	-	$t=R-\sqrt{R-\frac{d^{2}}{4}}$
Planar (Figure a)—The thickness of the layer, coatings is:	$h_{1}=\frac{1}{2}\left(\sqrt{4R^{2}-d^{2}}-\sqrt{4R^{2}-D^{2}}\right)$ $h_{1}=\frac{x*y}{2R}$	-
Cylindrical convex (Figure b) The thickness of the layer is:		-
Cylindrical concave (Figure c) The thickness of the layer is		-
Conical (Figure b) The thickness of the layer is		-
Spherical (Figure d) The thickness of the layer is:	$h_{1}=\frac{x\times y}{2}\left(\frac{1}{R_{b}}+\frac{1}{R}\right)$	-

**Table 2:** Methods of parametrical measurement of the coating on the spherical cut. All units appearing in the patterns and drawings are measurement in (µm).

	Trigonometric Relationship Measured Parameter	Diagram of the Deformed Areological System	Spherical Cut
1	Total penetration depth of the ball $T=R-\sqrt{R^{2}-r_{1}^{2}}$	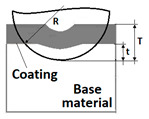	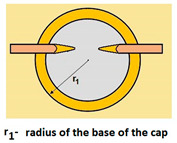
2	Coating thickness $h_{1}=\sqrt{R^{2}-r_{2}{}^{2}}-\sqrt{R^{2}-r_{1}{}^{2}}$	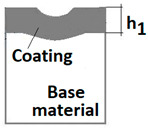	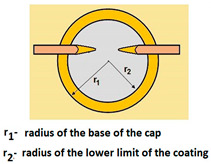
3	Scratch bottom depth $h_{s}=\sqrt{R^{2}-r_{s}{}^{2}}-\sqrt{R^{2}-r_{1}{}^{2}}$	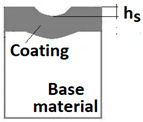	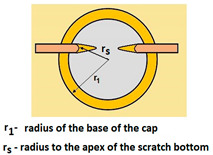
4	The depth of pressing the coating as measured from the surface $h_{d}=\sqrt{R^{2}-r_{d}{}^{2}}-\sqrt{R^{2}-r_{1}{}^{2}}$	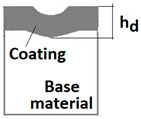	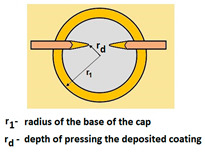
5	The depth of the coating pressing in measured from the surface of the base material $h_{db}=\sqrt{R^{2}-r_{d}{}^{2}}-\sqrt{R^{2}-r_{2}{}^{2}}$	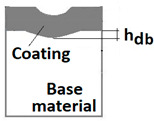	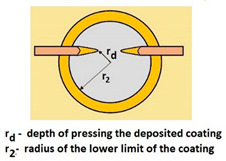
6	Thickness of the coating pressing under the scratch bottom $h_{cp}=\sqrt{R^{2}-r_{d}{}^{2}}-\sqrt{R^{2}-r_{s}{}^{2}}$	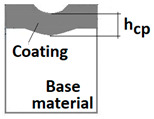	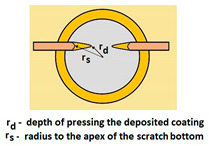
7	The height of the outflow of the coating material $h_{oc}=\sqrt{R^{2}-r_{1}{}^{2}}-\sqrt{R^{2}-r_{oc}{}^{2}}$	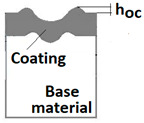	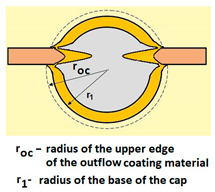
8	The height of the outflow of the base material $h_{os}=\sqrt{R^{2}-r_{2}{}^{2}}-\sqrt{R^{2}-r_{os}{}^{2}}$	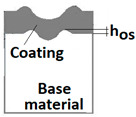	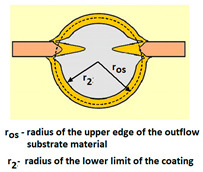

**Table 3:** Quantitative parameters *T*, *h_s_*, *h_oc_*, *h_os_* of the deformed coating structure geometry on spherical microsection obtained with the Racatest technique, Fn (N)-penetrator force range. Measurement of the scratch trace parameters on the left side (’) and right side (’’) of the spherical microsection.

Values	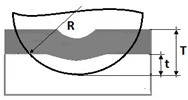	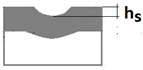		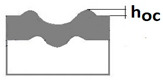		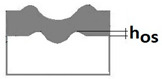	
Parametr	*T* (µm)	*h_s_*’ (µm)	*h_s_*’’ (µm)	*h_oc_*’ (µm)	*h_oc_*’’ (µm)	*h_os_*’ (µm)	*h_os_*’’ (µm)
1	4.0	−1.3	−1.9	-	-	-	-
2	5.0	−4.1	−4.1	1.1	0.9	1.3	0.9
3	6.5	−4.1	−4.7	2.2	1.9	1.9	1.9
4	7.9	−6.0	−6.2	2.2	2.5	2.4	3.4
5	9.0	−6.7	−6.7	3.6	4.1	3.5	3.8
5	10.9	−9.4	−10	4.2	-	4.8	-

## Data Availability

Data sharing is not applicable to this article.
